# Risk Factors of Aneurysmal Subarachnoid Hemorrhage Including Analysis by Sex: A Systematic Review and Meta-Analysis

**DOI:** 10.1212/WNL.0000000000213511

**Published:** 2025-03-28

**Authors:** Mariam Ali, Maaike J.A. van Eldik, Stijn Rietkerken, Jan W. Schoones, Nyika D. Kruyt, Gabriel J.E. Rinkel, Marieke J.H. Wermer, Sanne Peters, Ynte M. Ruigrok

**Affiliations:** 1 Department of Neurology, Leiden University Medical Center, the Netherlands;; 2 Department of Neurology and Neurosurgery, UMC Utrecht Brain Center, University Medical Center Utrecht, Utrecht University, the Netherlands;; 3 Directorate of Research Policy, Leiden University Medical Center, the Netherlands;; 4 Department of Neurology, University Medical Center Groningen, the Netherlands; and; 5 Julius Center for Health Sciences and Primary Care, University Medical Center Utrecht and Utrecht University, the Netherlands.

## Abstract

**Background and Objectives:**

A 2005 review identified smoking, hypertension, and excessive alcohol intake as the most important risk factors of aneurysmal subarachnoid hemorrhage (aSAH), but data on other factors remained inconclusive. While aSAH is more prevalent in female participants, evidence on sex differences and female-specific factors remains limited. Comprehensive identification of all risk factors, including potential sex differences and female-specific factors, is essential for improving prevention and accurately assessing aSAH risk. We aimed to determine whether there is now greater certainty around previously inconclusive risk factors, identify any new emerging factors, and explore sex differences in both established and emerging risk factors.

**Methods:**

We conducted a systematic review and meta-analysis of cohort and case-control studies on prevalent lifestyle exposures, following the Preferred Reporting Items for Systematic Reviews and Meta-Analyses statement. These exposures included smoking, hypertension, alcohol abuse, oral contraception, hormone replacement therapy, hypercholesterolemia, rigorous physical activity, lean body mass index, and diabetes. We calculated pooled sex-specific relative risks (RRs) and odds ratios (ORs) with 95% CIs for overall risk and female-to-male ratios of RRs (RRRs) and ORs (RORs) for sex comparisons.

**Results:**

We included 67 studies (34 cohort [8 with sex-specific data], 33 case-control [6 with sex-specific data]; n = 5,743,262; 57% female). A sex-specific association was found for current smoking (RRR 1.53, 95% CI 1.05–2.23), but not for hypertension (RRR 1.50, 95% CI 0.78–2.89) or excessive alcohol intake (RRR 0.46, 95% CI 0.13–1.63). Regular rigorous exercise (RR 0.74, 95% CI 0.53–1.04; OR 0.69, 95% CI 0.57–0.83) and diabetes (RR 0.75, 95% CI 0.55–1.02; OR 0.52, 95% CI 0.41–0.65) were associated with reduced risk, without sex-specific associations. Data on hypercholesterolemia (RR 1.24, 95% CI 0.97–1.58; OR 0.52, 95% CI 0.37–0.74) and lean BMI (RR 1.31, 95% CI 1.15–1.50; OR 1.39, 95% CI 0.74–2.60) were inconsistent and showed no sex-specific associations. Hormone replacement therapy (RR 1.03, 95% CI 0.72–1.48) and oral contraceptive use (RR 5.40, 95% CI 0.68–42.57) were limited to female patients, with current users compared with never users. Most studies contained potential sources of bias.

**Discussion:**

Current smoking, but not hypertension or excessive alcohol, has a stronger association with aSAH in female patients than in male patients. Regular exercise and diabetes are associated with a reduced risk, with no sex-specific associations. Data on female-specific factors remain inconsistent. Targeted smoking prevention may particularly benefit female patients. Large-scale studies are needed to clarify the role of female-specific factors in explaining the higher incidence of aSAH in female patients.

## Introduction

Rupture of an intracranial aneurysm leads to aneurysmal subarachnoid hemorrhage (aSAH), a severe type of stroke with substantial socioeconomic and health care burdens.^1^ Data suggest that female individuals have 1.61 times higher risk of aSAH than male individuals, although the reasons for this disparity remain unclear.^2^

Most aSAH cases are linked to lifestyle factors.^1^ A 2005 systematic review and meta-analysis identified smoking, hypertension, and excessive alcohol intake as the most important risk factors of aSAH. Data on other risk factors were less conclusive. Hypercholesterolemia (relative risk [RR] of 0.8 [95% CI 0.6–1.2] in longitudinal studies; odds ratio [OR] of 0.6 [95% CI 0.4–0.9] in case-control studies) and diabetes (RR 0.3, 95% CI 0–2.2; OR 0.7, 95% CI 0.5–0.8) were seemingly associated with a reduced risk while evidence for lean body mass index (BMI) (RR 0.3, 95% CI 0.2–0.4; OR 1.4, 95% CI 1.0–2.0) and rigorous exercise (RR 0.5, 95% CI 0.3–1.0; OR 1.2, 95% CI 1.0–1.6) was inconsistent.^3^ Two female-specific factors, hormone replacement therapy and oral contraceptive use, were assessed in female patients only, with current users compared with never and former users combined as the reference group. Hormone replacement therapy was potentially associated with reduced risk (RR 0.6, 95% CI 0.2–1.5; OR 0.6, 95% CI 0.4–0.8) while oral contraceptive use did not influence risk (RR 5.4, 95% CI 0.7–43.5; OR 0.8, 95% CI 0.5–1.3).^3^ Recent studies suggest sex-specific differences, particularly for smoking, although results are conflicting.^4^ Some studies indicate that female individuals who smoke may have a higher risk of aSAH compared with male individuals^5,6^ while others report no statistically significant sex differences.^7-9^

Given the publication of new research, we aim to provide an updated overview of the clinical risk factors of aSAH, assess whether new data offer more consistent and accurate insights into previously uncertain risk factors, and examine sex differences in both established and newly identified risk factors.

## Methods

This systematic review and meta-analysis followed the Preferred Reporting Items for Systematic Reviews and Meta-Analyses statement guidelines and was registered in PROSPERO (ID: CRD42023456329).^10^ We included all studies from the 2005 meta-analysis and conducted a systematic search in PubMed, Embase, Emcare, Web of Science, and the Cochrane Library (January 2005–March 2024).^3^ The search strategy, developed with an expert medical librarian, used terms related to (1) (aneurysmal) subarachnoid hemorrhage, (2) specific (vascular) risk factors, and (3) case-control and prospective cohort studies. Terms excluded case reports, reviews, and non-English studies. References of retrieved and review articles were also screened for additional studies until no further publications were identified. Full search strategy details are available online (eMethods 1 and 2).

### Selection Criteria

Inclusion criteria were as follows: (1) prospective cohort or (nested) case-control studies investigating aSAH from saccular aneurysm rupture; (2) provision of crude or adjusted effect estimates with 95% CIs for smoking, hypertension, alcohol use, oral contraceptive use, hormone replacement therapy, hypercholesterolemia, rigorous physical activity, lean BMI, and diabetes, or data allowing for their calculation; (3) overall (for male and female patients combined) or sex-specific data; (4) aSAH diagnosis confirmed by neuroimaging or International Classification of Diseases (ICD) codes; and (5) patients aged 18 years or older; and (6) articles in English (more details available online; eMethods 3). While this article focuses on sex differences, studies historically conflating sex and gender were included, and search terms encompassed both to ensure a comprehensive review.

### Definition and Classification of Risk Factors

We largely followed the 2005 meta-analysis,^3^ with some adjustments: (1) smoking status was categorized as current, former, and ever (current + former), using “never” as the reference; (2) for hormone replacement therapy and oral contraceptive use, current, former, and ever users were compared, using never users as the reference. This analysis was limited to female individuals. For the other factors, we use the definitions from the 2005 meta-analysis^3^: Alcohol consumption was classified as none, <150 g/wk, and ≥150 g/wk (assuming 12 g of ethanol per drink), with “no alcohol” as the reference. A lean BMI was defined as <22, with ≥22 as the reference. Hypertension (present vs absent), physical activity level (regular rigorous exercise vs no regular rigorous exercise), hypercholesterolemia (vs normal), and diabetes (present vs absent) followed the criteria from the original publications. When studies could not be categorized in line with these definitions, they were included in the systematic review but excluded from the meta-analysis.

### Study Selection, Data Extraction, and Risk-of-Bias Assessment

Two independent reviewers (M.A. and M.J.A.v.E.) conducted a two-stage screening process: review of titles and abstracts, followed by review of full-text articles. Data were extracted and recorded using a standardized data extraction form (available online; eMethods 4).

Quality assessment of the studies was performed with the Newcastle-Ottawa Scale for nonrandomized studies,^11^ which was adapted for the purpose of our study (details available online; eMethods 5). Disagreements in study selection, data extraction, and risk-of-bias assessment were resolved through consensus meetings involving a third reviewer (Y.M.R.).

### Statistical Analysis

For each study, we extracted ORs for case-control studies and RRs, rate ratios, or hazard ratios (HRs) with 95% CIs for cohort studies, or raw patient numbers for each risk factor. For studies with raw patient numbers, we calculated the standard error (SE) to determine the 95% CI using the following formula: SE = √[(1/a) + (1/b) + (1/c) + (1/d)], where “a” and “b” are the exposed and unexposed cases and “c” and “d” are the exposed and unexposed controls. Rate ratios and HRs were interpreted as RRs. We calculated overall estimates (male and female patients combined) and sex-specific estimates when data were available. Female-to-male ratios of RRs (RRRs) and ORs (RORs) were pooled using a random-effects meta-analysis. This approach weighted each estimate by the inverse variance of the log RRs or ORs. The pooled female-to-male ratios were then backtransformed to derive ratios for smoking, hypertension, alcohol use, hypercholesterolemia, physical activity, lean BMI, and diabetes. For female-specific factors, including hormone replacement therapy and oral contraceptive use, only RRs and ORs were calculated. If patients had multiple risk factors, they were included in the analysis of each risk factor. We used the most adjusted estimate available for each risk factor. If adjusted estimates were unavailable, crude effect estimates were pooled together with the adjusted estimates. A random-effects model was used to account for anticipated heterogeneity in patient characteristics and risk factor definitions. Heterogeneity was assessed using Higgins *I*^2^ statistics, with *I*^2^ of 25%–50% being moderate heterogeneity, 50%–75% substantial, and >75% considerable.^12^ Separate analyses were conducted for cohort and case-control studies, including forest plots for each risk factor. Funnel plots were used to evaluate publication bias when >10 studies reported on a risk factor (available online; eMethods 6). Statistical analyses were performed using R v.4.3.1.

### Standard Protocol Approvals, Registrations, and Patient Consents

Review board approval and informed consent were not required because this research used only published, deidentified data.

### Data Availability

Data not published within the article are available from the corresponding author on reasonable request. The data supplement is available online.

## Results

**Figure 1:**
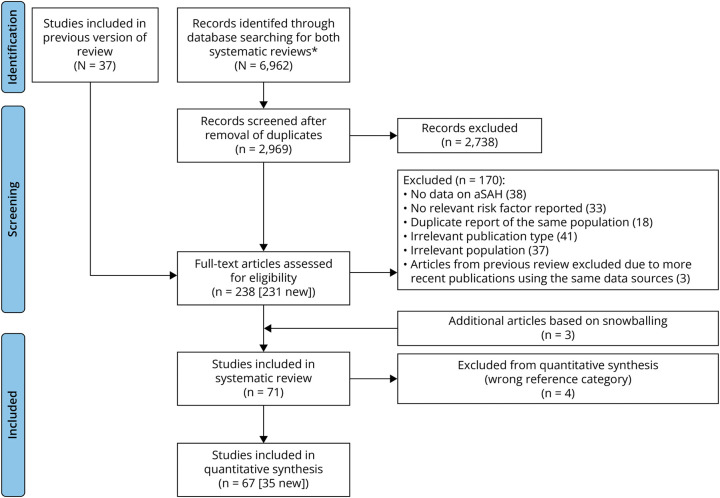
PRISMA Flow Diagram *The search identified 6,962 initial hits, including records for a systematic review on risk factors of unruptured intracranial aneurysms. aSAH = aneurysmal subarachnoid hemorrhage; PRISMA = Preferred Reporting Items for Systematic Reviews and Meta-Analyses.

In total, 2,969 records were identified from database search. In addition, 37 studies from the previous review were included. In total, 238 studies underwent full-text review. Seventy-one studies met the eligibility criteria (Figure 1),^6,7,13-47^ but 4 were excluded because their reference categories did not align with our predefined categories (available online; eMethods 7).^32,41,48,49^ Thus, 67 studies (34 longitudinal^6,8,9,27-31,33-40,42-47,50,e1-e11^ and 33 case-control studies^7,13-26,e12-e29^) were included in the meta-analysis^6-9,13-31,33-40,42-47,50,e1-e29^ while the systematic review included 36 longitudinal^6,8,9,27-47,50,e1-e11^ and 35 case-control studies^7,13-26,48,49,e12-e29^ (study characteristics summarized in eTables 1 and 2; supplemental references 1–38 regarding the eTables are available in the supplemental material).

These 71 studies encompassed 51 unique study populations, with 5,743,262 patients with aSAH (57% female; patient range per study 660–1,937,360). Forty-seven studies reported data on binary male-female sex (14 with sex-specific data),^6-8,13-25,27-31,33,34,36,37,39,42,44-47,49,e8,e9,e14-e19,e22-e24,e26-e29^ 17 on female-only,^32,35,38,40,41,43,48,e1,e3,e5,e6,e10,e12,e13,e20,e21,e25^ and 7 on male-only populations.^9,26,50,e2,e4,e7,e11^ The age range of participants was 18–90 years for studies including both sexes, 18–79 years for female-only studies, and 35–84 years for male-only studies. The study regions included 24 European, 20 Asian, 15 North American, 4 Oceanian, 3 Latin American, 1 Middle-Eastern, and 4 multicontinental studies (with contributions from Africa, Asia, Europe, and Latin America). Most studies were conducted in high-income countries, several in middle-income countries, and almost none exclusively in low-income countries.

### Risk-of-Bias Assessment

The overall quality of the studies varied. Most recruited patients from the general population and provided both crude and adjusted data on risk factors, suggesting a generally lower risk of bias. However, differences in methods for confirming aSAH and assessing risk factors were present: some studies relied on neuroimaging and angiography, classified as low risk of bias, whereas others used less precise methods, such as ICD codes or medical records, which were classified as higher risk of bias. Most studies contained 1 or 2 sources of bias, with only 1 study fulfilling our criteria for high methodological quality (available online; eTables 3 and 4).^38^ Full results, including funnel plots, are available online (eAppendix 1 and eFigure 1).

### Meta-Analysis

**Figure 2:**
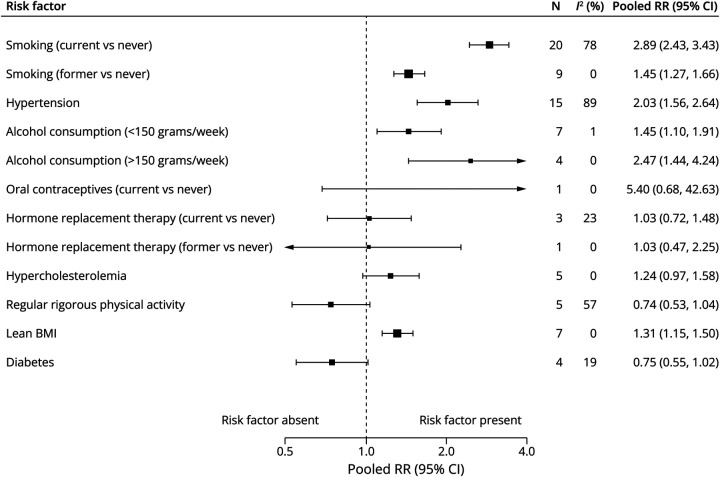
Summary Forest Plot of Cohort Studies BMI = body mass index; N = number of studies; RR = relative risk.

**Figure 3:**
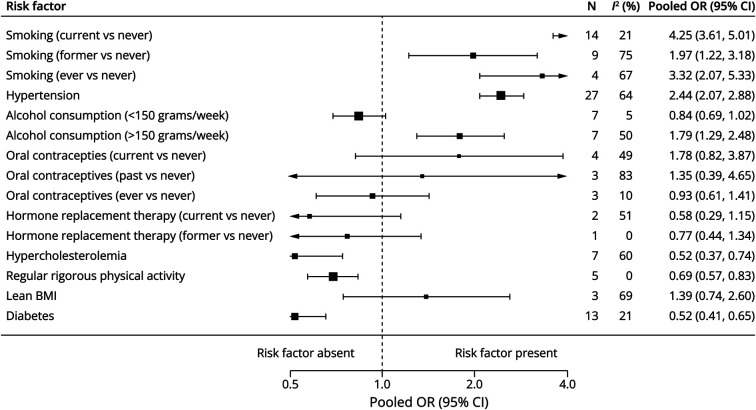
Summary Forest Plot of Case-Control Studies BMI = body mass index; N = number of studies; OR = odds ratio.

**Figure 4:**
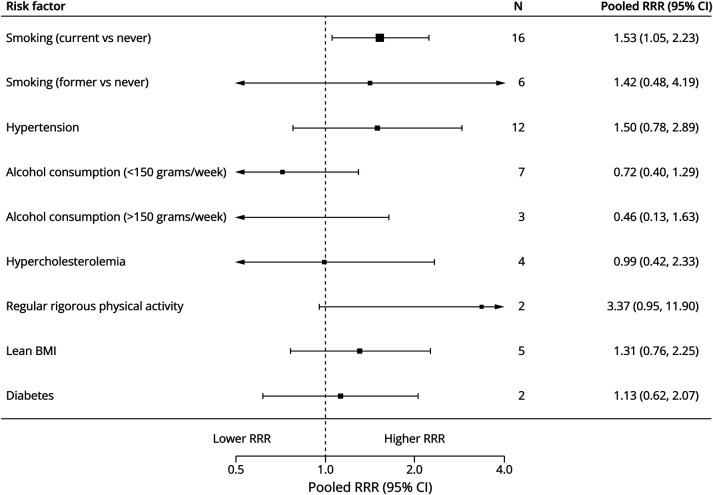
Summary Forest Plot of Female-to-Male RRRs BMI = body mass index; N = number of studies; RRR = ratio of relative risk.

**Figure 5:**
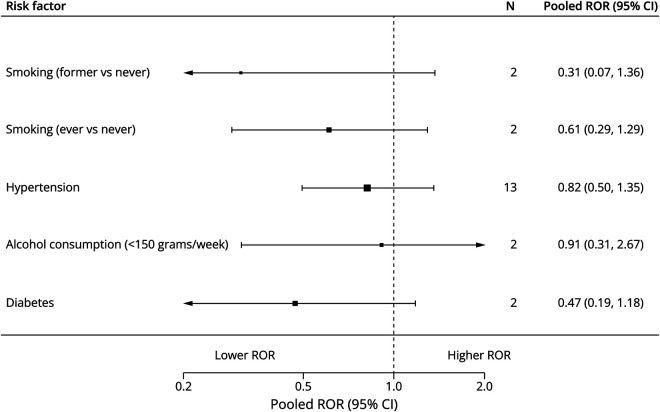
Summary Forest Plot of Female-to-Male RORs N = number of studies; ROR = ratio of odds ratio.

Individual forest plots for each risk factor are available in the online (eFigure 2–16). Figure 2 shows the summary forest plots for cohort studies, Figure 3 for case-control studies, Figure 4 for RRRs, and Figure 5 for RORs.

#### Smoking

Current smoking (vs never) was associated with higher aSAH risk in both cohort studies (pooled RR 2.89, 95% CI 2.43–3.43, *I*^2^ = 78%, 20 studies, 3,817,992 patients) and case-control studies (OR 4.25, 95% CI 3.61–5.01, *I*^2^ = 21%, 14 studies, 11,979 patients). The association was stronger in female patients compared with male patients (RRR 1.53, 95% CI 1.05–2.23; Table 1). Former smoking was also associated with higher aSAH risk (RR 1.45, 95% CI 1.27–1.66, *I*^2^ = 0%, 9 studies, 808,420 patients; OR 1.97, 95% CI 1.22–3.18, *I*^2^ = 75%, 9 studies, 5,507 patients), but without a statistically significant sex-specific association (RRR 1.42, 95% CI 0.48–4.17; ROR 0.31, 95% CI 0.07–1.35). Ever smoking was assessed only in case-control studies and showed a similar association (OR 3.32, 95% CI 2.07–5.33, *I*^2^ = 67%, 4 studies, 2,471 patients).

**Table 1:** Effect Sizes of Risk Factors Stratified by Sex and Study Design

Risk factor	Female	Male	Total^a^	Female-to-male RRRs	Female-to-male RORs
Smoking (vs never)					
Cohort studies					
Current (W: n = 8; M: n = 8)	3.52 (2.61–4.75)	2.31 (1.84–2.92)	2.89 (2.43–3.43)	1.53 (1.05–2.23)	—
Former (W: n = 3; M: n = 3)	1.56 (0.73–3.32)	1.10 (0.51–2.37)	1.45 (1.27–1.66)	1.42 (0.48–4.17)	—
Case-control studies					
Current (W: n = 1)	5.70 (1.51–21.53)	—	4.25 (3.61–5.01)	—	—
Former (W: n = 1; M: n = 1)	0.49 (0.16–1.50)	1.57 (0.61–4.03)	1.97 (1.22–3.18)	—	0.31 (0.07–1.35)
Ever (W: n = 1; M: n = 1)	2.77 (1.52–5.04)	4.54 (2.87–7.21)	3.32 (2.07–5.33)	—	0.61 (0.29–1.30)
Hypertension (vs none)					
Cohort studies (W: n = 6; M: n = 6)	2.67 (1.56–4.56)	1.78 (1.22–2.61)	2.03 (1.56–2.64)	1.50 (0.78–2.89)	—
Case-control studies (W: n = 8; M: n = 5)	2.45 (1.68–3.58)	2.99 (2.15–4.16)	2.44 (2.07–2.88)	—	0.82 (0.50–1.36)
Alcohol intake (vs none)					
Cohort studies					
<150 g/wk (W: n = 4; M: n = 3)	1.31 (0.94–1.82)	1.81 (1.12–2.92)	1.45 (1.10–1.91)	0.72 (0.40–1.29)	—
≥150 g/wk (W: n = 1; M: n = 2)	1.86 (0.79–4.40)	4.02 (1.60–10.08)	2.47 (1.44–4.24)	0.46 (0.13–1.63)	—
Case-control studies					
<150 g/wk (W: n = 1; M: n = 1)	0.71 (0.39–1.28)	0.78 (0.32–1.91)	0.84 (0.69–1.02)	—	0.91 (0.31–2.66)
≥150 g/wk (M: n = 1)	—	1.48 (0.59–3.72)	1.79 (1.29–2.48)	—	—
Hypercholesterolemia (vs normal)					
Cohort studies (W: n = 1; M: n = 3)	1.20 (0.64–2.24)	1.21 (0.68–2.18)	1.24 (0.97–1.58)	0.99 (0.42–2.33)	—
Case-control studies	—	—	0.52 (0.37–0.75)	—	—
Regular rigorous physical activity (vs no regular rigorous exercise)					
Cohort studies (W: n = 1; M: n = 1)	0.91 (0.71–1.16)	0.27 (0.10–0.76)	0.74 (0.53–1.04)	3.37 (0.93–11.60)	—
Case-control studies	—	—	0.69 (0.57–0.83)	—	—
Lean BMI <22 (vs ≥ 22)					
Cohort studies (W: n = 2; M: n = 3)	1.70 (1.13–2.56)	1.30 (0.92–1.84)	1.31 (1.14–1.49)	1.31 (0.76–2.24)	—
Case-control studies (W: n = 1)	0.74 (0.35–1.54)	—	1.39 (0.74–2.58)	—	—
Diabetes (vs none)					
Cohort studies (W: n = 1; M: n = 1)	0.93 (0.59–1.47)	0.82 (0.55–1.22)	0.75 (0.55–1.02)	1.13 (0.62–2.08)	—
Case-control studies (W: n = 1; M: n = 1)	0.26 (0.13–0.52)	0.55 (0.30–1.02)	0.52 (0.41–0.65)	—	0.47 (0.19–1.19)
Oral contraceptive use (vs never)					
Cohort studies					
Current	5.40 (0.69–42.57)	—	—	—	—
Former	—	—	—	—	—
Case-control studies					
Current	1.78 (0.82–3.87)	—	—	—	—
Former	1.35 (0.39–4.62)	—	—	—	—
Ever	0.93 (0.61–1.41)	—	—	—	—
Hormone replacement therapy (vs never)					
Cohort studies					
Current	1.03 (0.72–1.48)	—	—	—	—
Former	1.03 (0.47–2.25)	—	—	—	—
Case-control studies					
Current	0.58 (0.29–1.14)	—	—	—	—
Former	0.77 (0.44–1.33)	—	—	—	—

Abbreviations: ROR = ratio of odds ratio; RRR = ratio of relative risk.

a Totals represent pooled estimates from all studies included in the forest plot. For studies that reported risk factors separately for male and female patients, sex-specific estimates were calculated.

#### Hypertension

Hypertension was associated with increased aSAH risk in both cohort studies (RR 2.03, 95% CI 1.56–2.64, *I*^2^ = 89%, 15 studies, 2,441,026 patients) and case-control studies (OR 2.44, 95% CI 2.07–2.88, *I*^2^ = 64%, 27 studies, 8,322,556 patients), but no statistically significant sex-specific association was observed (RRR 1.50, 95% CI 0.78–2.89; ROR 0.82, 95% CI 0.50–1.36).

#### Alcohol Consumption

Moderate alcohol consumption (<150 g/wk) (vs no alcohol) was modestly associated with increased aSAH risk in cohort studies (RR 1.45, 95% CI 1.10–1.91, *I*^2^ = 1%, 7 studies, 299,443 patients) but not in case-control studies (OR 0.84, 95% CI 0.69–1.02, *I*^2^ = 5%, 7 studies, 1,449,394 patients), with no statistically significant sex-specific association (RRR 0.72, 95% CI 0.40–1.29; ROR 0.91, 95% CI 0.31–2.66). Excessive alcohol intake (≥150 g/wk) (vs no alcohol) was linked to higher aSAH risk in both cohort studies (RR 2.47, 95% CI 1.44–4.24, *I*^2^ = 0%, 4 studies, 196,412 patients) and case-control studies (OR 1.79, 95% CI 1.29–2.48, *I*^2^ = 50%, 7 studies, 4,418 patients), again with no statistically significant sex-specific association (RRR 0.46, 95% CI 0.13–1.63).

#### Lean BMI

Lean BMI (<22) was associated with increased aSAH risk in cohort studies (RR 1.31, 95% CI 1.15–1.50, *I*^2^ = 0%, 7 studies, 1,044,285 patients), but not in case-control studies (OR 1.39, 95% CI 0.74–2.60, *I*^2^ = 69%, 3 studies, 1,437 patients), with no statistically significant sex-specific association (RRR 1.31, 95% CI 0.76–2.24).

#### Physical Activity

There was no statistically significant association between regular rigorous physical activity and aSAH risk in cohort studies (RR 0.74, 95% CI 0.53–1.04, *I*^2^ = 57%, 5 studies, 1,370,696 patients). However, case-control studies showed a statistically significant association (OR 0.69, 95% CI 0.57–0.83, *I*^2^ = 0%, 5 studies, 2,579,908 patients), with no statistically significant sex-specific association (RRR 3.37, 95% CI 0.93–11.60).

#### Diabetes

There was no statistically significant association between diabetes and aSAH risk in cohort studies (RR 0.75, 95% CI 0.55–1.02, *I*^2^ = 19%, 4 studies, 992,545 patients). However, case-control studies showed a statistically significant association (OR 0.52, 95% CI 0.41–0.65, *I*^2^ = 21%, 13 studies, 5,371,888 patients), with no statistically significant sex-specific association (RRR 1.13, 95% CI 0.62–2.08; ROR 0.47, 95% CI 0.19–1.19).

#### Hypercholesterolemia

Hypercholesterolemia showed inconsistent associations, with a nonstatistically significant increase in risk in cohort studies (RR 1.24, 95% CI 0.97–1.58, *I*^2^ = 0%, 5 studies, 245,383 patients), but was associated with a reduced risk in case-control studies (OR 0.52, 95% CI 0.37–0.74, *I*^2^ = 60%, 7 studies, 3,065,754 patients). The RRR and ROR did not suggest a sex-specific association.

#### Female-Specific Factors: Hormone Replacement Therapy and Oral Contraceptive Use

Neither current hormone replacement therapy (RR 1.03, 95% CI 0.72–1.48, *I*^2^ = 23%, 3 studies, 1,159,991 patients; OR 0.58, 95% CI 0.29–1.15, *I*^2^ = 51%, 2 studies, 863 patients) nor current oral contraceptive use (RR 5.40, 95% CI 0.68–42.57, *I*^2^ = 0%, 1 study, 17,032 patients; OR 1.78, 95% CI 0.82–3.87, *I*^2^ = 49%, 4 studies, 599,979 patients) had a statistically significant association with aSAH risk.

## Discussion

The association between aSAH and current smoking is stronger in female patients than in male patients, whereas associations with hypertension and excessive alcohol are similar for both sexes. Diabetes and regular rigorous physical activity are associated with a reduced risk of aSAH in case-control studies, but this association was not observed in cohort studies. With 35 more studies included compared with the 2005 meta-analysis, findings on regular rigorous exercise are now more consistent and data on diabetes are more reliable. Associations with hypercholesterolemia, lean BMI, and female-specific factors such as hormone replacement therapy and oral contraceptive use remain uncertain because of differences between pooled ORs and RRs, but overall, no statistically significant associations were observed. However, these results should be interpreted with caution because of potential biases, heterogeneity, and limited availability of sex-specific data. There is also variation in the number and type of adjustment factors between studies, including age, as well as a predominance of studies from high-income countries with limited representation from low-income and middle-income countries. Smoking, hypertension, and excessive alcohol consumption remain the most important risk factors of aSAH.

Our study, with nearly twice as many studies as the 2005 meta-analysis,^3^ reaffirms smoking, hypertension, and excessive alcohol intake as the most important risk factors and provides more precise estimates. The 2005 meta-analysis reports inconsistent findings for lean BMI and rigorous exercise. Our analysis offers improved insights into these associations: lean BMI is associated with increased risk (although borderline statistically significant in case-control studies), and rigorous exercise is associated with a reduced risk (although borderline statistically significant in cohort studies). The inverse association between diabetes and aSAH risk observed in 2005 is confirmed and now more reliable (previous RR 0.3, 95% CI 0–2.2; OR 0.7, 95% CI 0.5–0.8; now RR 0.75, 95% CI 0.55–1.02; OR 0.52, 95% CI 0.41–0.65). However, the association with hypercholesterolemia remains inconsistent, with case-control studies suggesting lower risk and cohort studies showing no statistically significant relationship. Unlike the 2005 analysis, our study finds no statistically significant association between hormone replacement therapy and aSAH. A 2019 systematic review and meta-analysis, which focuses on studies specifically examining sex differences, found no sex-specific associations for hypertension and smoking.^5^ By contrast, our study suggests a potential sex-specific association for hypertension in female participants, although it is not statistically significant. This difference may be due to the exclusion of many studies in the 2019 review that we include because their analysis only considers risk factors stratified by sex or those with an interaction term between sex and aSAH risk factors.

The observed sex differences in risk factors may be explained by biological, hormonal, and behavioral differences between male and female patients. For instance, female patients might be more susceptible to the vascular effects of smoking. One hypothesis is that smoking lowers estrogen levels, which could influence vascular profiles and partly explain this differential impact. This is especially relevant because the incidence of aSAH in female individuals increases significantly after age 50 compared with male individuals, a time when estrogen levels naturally decline.^4^

The association between low BMI and an increased risk of aSAH has also been reported in other studies on hemorrhagic stroke, including ICH and aSAH-specific research.^8,44,e30-e33^ This relationship may be confounded by weight loss associated with high alcohol consumption and smoking.^44,e30,e34,e35^ Of the 8 articles describing low BMI included in our meta-analysis, only 4 studies adjusted for alcohol overuse and smoking.

The strengths of our systematic review and meta-analysis include the large number of studies, encompassing nearly 6,000,000 patients, and the comprehensive evaluation of a wide range of risk factors. We conduct subgroup analyses by study type to assess the impact on effect estimates and heterogeneity, as well as by specific risk factors, such as current, former, and ever smoking. In addition, we provide a detailed risk-of-bias assessment and use funnel plots to address heterogeneity, to better interpret the results.

Our study also has several limitations that should be considered. First, significant heterogeneity among the included studies hampers interpretation of several pooled effect estimates. This heterogeneity stems from factors such as differences in study design, population characteristics, and methods of aSAH diagnosis. Substantial differences in heterogeneity between cohort and case-control studies for several risk factors suggest that study design plays a key role, although no consistent pattern indicates that 1 design consistently has lower heterogeneity. Second, several sources of bias might be present, including the following: (1) publication bias, suggested by funnel plot asymmetry for several risk factors, indicating a tendency to publish studies with statistically significant findings; (2) selection bias from restricted populations, affecting external validity; (3) information bias due to inconsistent aSAH and risk factor data collection; (4) recall and misclassification biases from varying risk factor definitions; and (5) investigation bias from different evaluation methods, which may lead to discrepancies and inaccurate effect size estimates.

In addition, we analyze only 1 risk factor at a time, potentially overlooking interrelationships and cumulative effects of multiple risk factors, which may lead to underestimating their combined impact on aSAH risk. Individuals with multiple risk factors are often at a higher risk, but this was not assessed in our analysis. Furthermore, we analyze risk factors dichotomously (e.g., hypertension yes/no and smoking current/former/never), which limits our ability to capture differences in exposure levels. For example, male patients may smoke more heavily than female patients, but our analysis treats them equally.^e36,e37^

In addition, not all studies account for age or other residual confounding factors, with considerable variation in adjustments used, complicating reliability. Third, although 67 studies are included, the number of studies available for some risk factors, such as oral contraceptive use, hormone replacement therapy, and lean BMI, is too small for robust conclusions. In addition, most studies do not report data separately by sex, limiting sex-specific analyses and underscoring the need for future research. Furthermore, race-related and ethnicity-related differences were not evaluated, which could moderate these findings. Fourth, some studies combine risk factors or use reference categories that do not fit our classification system, making them ineligible for inclusion in the meta-analysis.^7,15,26,34,38,48,49,e17,e29^ Furthermore, changes in oral contraceptive dosages over time—such as the shift from high-dose to low-dose formulations—could not be accounted for because most studies lacked dosage-specific data. This may affect the interpretation of findings on exogenous estrogen exposure. Fifth, most studies provided both crude and adjusted data on risk factors. When adjusted estimates were unavailable, crude estimates were pooled with adjusted ones. This approach may have introduced bias because crude estimates do not account for confounding factors.

Female patients have a statistically significant stronger association with current smoking, suggesting that targeted prevention efforts may be particularly beneficial for this group. Because smoking, hypertension, and excessive alcohol consumption remain the most important risk factors of aSAH, information on these—including the sex-specific effect of smoking—should be incorporated into risk assessments, such as the PHASES score, to guide prevention strategies and improve risk management.^e38^ Regular rigorous exercise and diabetes are associated with a reduced risk of aSAH, whereas the associations with lean BMI, hypercholesterolemia, and female-specific factors such as hormone replacement therapy and oral contraceptive use remain unclear because of inconsistent findings. Because the sex-specific risk of smoking alone does not fully explain the higher incidence of aSAH in female patients, large-scale studies are needed to clarify the role of female-specific factors in aSAH.

## Glossary

aSAH: aneurysmal subarachnoid hemorrhage
BMI: body mass index
HR: hazard ratio
ICD: International Classification of Diseases
OR: odds ratio
ROR: ratio of OR
RR: relative risk
RRR: ratio of RR
SE: standard error
